# Migration of Myeloid Cells during Inflammation Is Differentially Regulated by the Cell Surface Receptors Slamf1 and Slamf8

**DOI:** 10.1371/journal.pone.0121968

**Published:** 2015-03-23

**Authors:** Guoxing Wang, Boaz J. van Driel, Gongxian Liao, Michael S. O’Keeffe, Peter J. Halibozek, Jacky Flipse, Burcu Yigit, Veronica Azcutia, Francis W. Luscinskas, Ninghai Wang, Cox Terhorst

**Affiliations:** 1 Department of Medicine, Division of Immunology, Beth Israel Deaconess Medical Center, Harvard Medical School, Boston, Massachusetts, United States of America; 2 Department of Pathology, Center for Excellence in Vascular Biology, Brigham and Women's Hospital, Harvard Medical School, Boston, Massachusetts, United States of America; The Ohio State University, UNITED STATES

## Abstract

Previous studies have demonstrated that the cell surface receptor Slamf1 (CD150) is requisite for optimal NADPH-oxidase (Nox2) dependent reactive oxygen species (ROS) production by phagocytes in response to Gram- bacteria. By contrast, Slamf8 (CD353) is a negative regulator of ROS in response to Gram+ and Gram- bacteria. Employing *in vivo* migration after skin sensitization, induction of peritonitis, and repopulation of the small intestine demonstrates that *in vivo* migration of Slamf1^-/-^ dendritic cells and macrophages is reduced, as compared to wt mice. By contrast, *in vivo* migration of Slamf8^-/-^ dendritic cells, macrophages and neutrophils is accelerated. These opposing effects of Slamf1 and Slamf8 are cell-intrinsic as judged by *in vitro* migration in transwell chambers in response to CCL19, CCL21 or CSF-1. Importantly, inhibiting ROS production of Slamf8^-/-^ macrophages by diphenyleneiodonium chloride blocks this *in vitro* migration. We conclude that Slamf1 and Slamf8 govern ROS–dependent innate immune responses of myeloid cells, thus modulating migration of these cells during inflammation in an opposing manner.

## Introduction

Slamf1 (CD150, SLAM) and Slamf8 (CD353, BLAME) belong to the nine member Signaling Lymphocyte Activating Molecule (SLAM) family of hematopoietic cell surface receptors, which regulate a variety of immune responses, including T-cell activation, antibody generation, cytokine production, and natural killer-T-cell (NKT) development [1]. Slamf1, 3, 5, 6, and 7 are homophilic receptors and Slamf4 and Slamf2 are co-ligands [1–4]. The T-cell co-stimulatory molecule Slamf1 signals in part through a specific association with the SLAM associated protein (SAP) [5]. In addition, in macrophages Slamf1 acts as a microbial sensor, which in response to *Escherichia coli* interacts with a Beclin-1> UVRAG>Vps34 complex. The Slamf1>Beclin-1>UVRAG>Vps34 complex converts phosphatidyl-inositol to phosphatidyl-inositol-3’phosphate (PI_3_P), which activates the reactive oxygen producing enzyme complex Nox2 and promotes phagosome maturation [6, 7]. As both of these bactericidal processes are involved in the killing of Gram- bacteria, removal of the attenuated *S*. *Typhumurium Sseb*- in Slamf1^-/-^ mice is impaired [6]. Slamf1 and human SLAM are also expressed by neutrophils, monocytes, and activated dendritic cells (DCs)[8].

The cell surface glycoprotein Slamf8, as well as human SLAMF8 (BLAME), is expressed by a variety of myeloid cells such as neutrophils, macrophages, monocytes and DCs upon encountering Gram- or Gram+ bacteria, LPS or IFN-γ [9, 10]. In contrast to Slamf1, Slamf8 negatively regulates ROS production by inhibiting Nox2 activity and conversely Nox2 activity of Slamf8^-/-^ macrophages is increased upon exposure to bacteria. One explanation could be that Slamf8 regulate protein kinase C activity, which is increased in Slamf8^-/-^ macrophages causing an enhanced phosphorylation of p40phox, in turn leading to greater Nox2 activity [10].

The classic paradigm that production of ROS by phagocytes is instrumental in their bactericidal activity is complemented by more recent studies that have established a role in the activation of many signaling pathways and the regulation of antigen cross-presentation [11, 12]. Perhaps more importantly, ROS also affects cell adhesion and migration [13, 14]. Recently two other groups independently reported that Nox2 regulates CSF-1 mediated macrophage and microglial chemotaxis [15, 16]. In support of this notion are our observations that infiltration of Slamf1^-/-^ macrophages and monocytes to the sites of inflammation is markedly reduced in two models of enterocolitis and in peritonitis [17].

The present study evaluates the respective roles of Slamf1 and Slamf8 in migration of dendritic cells (DCs), macrophages, and neutrophils by employing *in vivo* and *in vitro* assays. Not only does a correlation exist between the level of ROS production and altered migration in a cell-intrinsic fashion, the use of a Nox2 inhibitor prevents *in vitro* migration. As Slamf8 is a homophilic cell surface receptor, which is expressed on endothelial cells, we postulate that Slamf8 may control migration by adhesion to the lymphatic capillaries. Similarly, expression of Slamf8 by fibroblastic reticular cells (FRC) [18] suggests that this receptor may play a role in migration of DCs along FRC-containing conduits inside the lymph nodes.

## Materials and Methods

### Mice

Slamf1^-/-^ and Slamf8^-/-^ *Balb/c* mice were described previously [5, 10]. Age and sex matched wt *Balb/c* mice were purchased from The Jackson Laboratory (Bar Harbor, ME, USA). All animals were maintained under specific pathogen-free conditions at the Center for Life Science animal facility of Beth Israel Deaconess Medical Center (BIDMC) and were used at 8–12 weeks of age. The experiments were performed according to the guidelines of the Institutional Animal Care and Use Committee (IACUC) at BIDMC.

### *In vivo* assay for migration of skin DC-FITC painting assay

Experiments were performed as described [19]. In brief, the dorsal skin of individual mice that were anesthetized with isoflurane was shaved, followed by application of 400μL of 10mg/mL FITC dissolved in 1:1 acetone/dibutylphthalate (Sigma-Aldrich). After 24 hours, mouse inguinal and axillary lymph nodes were isolated by digestion at 37°C for 1h with a cocktail of 100U/ml DNase I (fraction IX; Sigma-Aldrich) and 1.6 mg/mL collagenase (CLS4; Worthington Biochemical). In the experiment using Slamf8-Fc fusion, 100μg of Slamf8-Fc fusion protein or human IgG-Fc (Jackson ImmunoResearch Laboratories) was intraperitoneally injected 3 hours before 200μL FITC application. Single-cell suspensions were stained and analyzed by flow cytometry.

### *In vivo* Thioglycollate broth-induced peritonitis

Wt, Slamf1^-/-^ and Slamf8^-/-^ mice were intraperitoneally injected with 2mL of sterile 4% thioglycollate broth. Four hours or four days later, mice were euthanized. The cells from the peritoneal cavity were washed out with 10mL of RPMI medium and stained for FACS.

### *In vivo* Anti-CD3 induced myeloid cell migration model

20μg of purified αCD3ε (145–2C11) (Biolegend, San Diego, CA) or Mouse IgG2b (Biolegend, San Diego, CA) were intraperitoneally injected. Three or five days later the mice were euthanized. Lamina propria cells were isolated and analyzed by flow cytometry [20].

### Nox2 lucigenin assays

Nox2 assays using lucigenin (Sigma-Aldrich, St. Louis, MO) were done using a standard Glomax luminometer (Promega, Madison, WI) after exposure to heat inactivated *E*.*coli* F18 bacteria (multiplicity of infection [MOI] 100) or phorbol myristate acetate (PMA) (1μg/ml) [6].

### Intracellular ROS detection

Intracellular ROS was quantified by using CM-H_2_DCFDA (Life Technologies, C-6827) based on the manufacturer's protocol. CM-H_2_DCFDA was added to RPMI 1640 medium at a final concentration of 10μM. Thioglycollate-elicited macrophages were incubated with CM-H_2_DCFDA at 37°C for 1 hour. After incubation, the macrophages were incubated with heat inactivated *E*.*coli* F18 bacteria (multiplicity of infection [MOI] 100). Fluorescence was measured every 5 minutes by flow cytometry for 2 hours.

### *In vitro* Chemotaxis assay

Ear skin was collected and digested for 2 hours with DNase (15μl from 10 mg/ml stock, Sigma) and 500μL Liberase (Roche) in RPMI 1640 at 37°C in a shaking incubator. After filtration through a 70μm cell strainer, single cell suspensions were obtained. CD11c^+^ cells were further isolated using CD11c MicroBeads (Miltenyi Biotec) in a positive selection column. Chemotaxis of skin DCs was measured using a polycarbonate filter with 5μm pores in 96-well transwell chambers (Neuro Probe, Gaithersburg MD). 30μL chemotaxis media was added to the lower chamber, and 2.5 × 10^4^ DCs were added to the upper chamber and incubated for 8 hour at 37°C with 5% CO_2_.

*In vitro* transwell (5μm pore) migration analysis for macrophages was performed using a 96-well migration chamber (NeuroProbe, Gaithersburg MD). The lower wells contained recombinant murine CSF-1 (30ng/mL), and 5.0 × 10^4^ Thio-macrophages were added to the upper well to migrate for 150 minutes in a humidified chamber (5% CO_2_, 37°C). In some experiments diphenyleneiodonium chloride (DPI) (5μM) was added to cell suspension 15 minutes prior to migration. Migrating cells were counted according to the manufacturer’s protocol by flow cytometry, using counting beads.

### Generation of mouse Slamf8-Fc and Slamf1-Fc recombinant soluble protein

The murine Slamf8-Fc and Slamf1-Fc fusion protein containing the Fc region of human IgG1 was obtained by inserting the sequence corresponding to the extracellular domain (including the signal peptides and two extracellular domains) of Slamf8 or Slamf1 into the mammalian expression vector pcDNA4/myc-HisC (Invitrogen). The construct expressing Slamf8-Fc was transfected in NS-1 myeloma cells and the construct expressing Slamf1-Fc was transfected in 293F cells. Stable Slamf8-Fc or Slamf1-Fc expressing cells were selected with *zeocin* and expanded for large-scale culture. The supernatants containing the fusion proteins were purified with a protein G agarose bead column from the supernatant.

### Slamf8-Fc fusion protein binding assay

Mouse Slamf8 cDNA were cloned into pcDNA4.0 Myc/his vector (Life Technology, Carlsbad, CA, USA). HEK293 cells were transfected with mouse Slamf1 or Slamf8 cDNAs using FuGENE 6 Transfection Reagent (Roche). 24 hours after transfection, 10μg of Fc fusion proteins Slamf1-Fc or Slamf8-Fc were incubated with 1×10^6^ transfected HEK293 cells on ice for 60 minutes, followed by incubation with biotin-conjugated goat anti-human IgG Fc (Life Technologies) and then streptavidin-PE. Binding was assessed by flow cytometry.

### Flow Cytometry

Macrophages, DCs and neutrophils were incubated with Fc-blocker at 4°C for 20 minutes. Samples were stained with; MHC class II (Biolegend), CD11c (BD Bioscience), CD11b (BD Bioscience), F4/80 (eBioscience), Ly6G (Biolegend), CD115 (Biolegend) and CCR7 (Biolegend), CD103 (Biolegend), Ly6C (eBioscience), CD45.2 (Biolegend) on ice for 30 min. Dead cells were visually excluded by DAPI (Roche). The cells were acquired on a BD LSRII flow cytometer and the data analysis was performed by FlowJo software (Trees Star Inc. Ashland, OR).

### Preparation of Mouse Heart Endothelial Cells

Mouse heart endothelial cells (MHEC) were prepared from the heart of newborn mice (7–9 days old) as described [21]. 100ng/ml of TNF-α was used to stimulate endothelial cells for 24 hours, and the cell RNA was isolated.

### RNA isolation and real-time PCR (qPCR)

Bone marrow derived DCs and peritoneal Thio-neutrophils total RNA was isolated using TRIzol (Invitrogen). qPCR was performed and analyzed on the 7500 FAST Real-Time PCR System (Applied Biosystems). Slamf8 and the Eukaryotic 18S ribosomal RNA Endogenous Control TaqMan probes were purchased from Life Technologies (Carlsbad, CA, USA). Slamf8: Forward: 5’-CCTGGCTGGTCTCTTTGGG-3’; Reverse 5’-CGTCAGTGCAAGCATCCTTC-3’; Probe: 5’-CACCATGGCCTCTGCTCAGGGAAG-3’. Human SLAMF8: Forward: 5’-GCTTCCAAGTCCGTGAGGC-3’; Reverse 5’-AAAACGTGGCCAGGAGCTC-3’. The relative gene-specific fold change, normalized to 18S rRNA, was calculated using the 2−ΔΔct method and expressed relative to untreated wt levels.

### Western Blot

Thio-Macrophages were stimulated with IFN-γ (10ng/ml) plus LPS (100ng/ml) overnight. Slamf8 protein expression was detected with Sheep anti-mouse Slamf8 Polyclonal antibody (R&D System, AF4156) by Western Blot as described [7].

### Statistical analysis

The Prism 5.0 software (GraphPad, San Diego, CA, USA) was used to analyze the data. Results are reported as mean ± SEM. All the statistical comparisons were performed using the 2-tailed Student’s t test. Values of p < 0.05 were considered to be statistically significant.

### Ethics statement

The murine protocols used in this study were approved by the BIDMC IACUC (Protocol 020–2012), which is accredited by the Association for Assessment and Accreditation of Laboratory Animal Care International (AAALAC). All efforts were made to minimize animal suffering, including the use of isoflurane when appropriate, i.e., in the skin sensitization assay. Euthanasia was performed by CO_2_ in an IACUC-approved setup. The use of PBMCs was approved by the Institutional Review Board of Beth Israel Deaconess Medical Center.

## Results

### Slamf8 negatively regulates *in vivo* migration of DCs, whilst Slamf1 is a positive regulator

**Fig 1:**
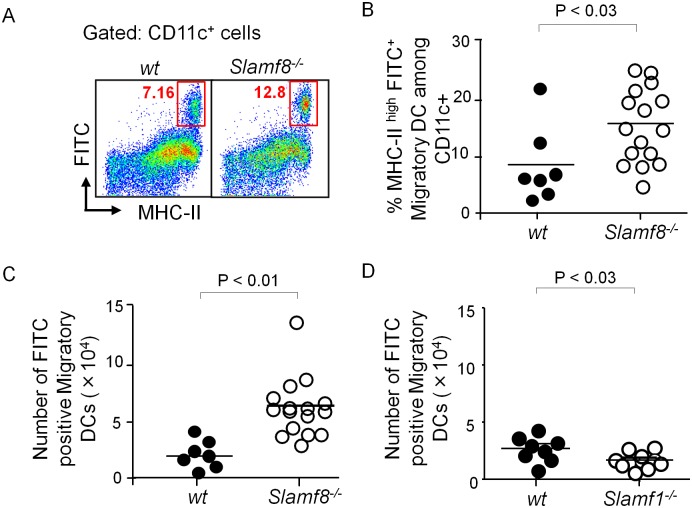
Slamf8 negatively and Slamf1 positively affects DC migration from the skin to draining lymph nodes after Hapten-FITC sensitization. **(A)** Representative dot plots (gated on CD11c^+^ cells) and **(B)** percentages of migratory MHC-II^hi^ FITC^+^ DCs in the draining LNs of wt and Slamf8^-/-^ mice, 24 h after painting dorsal skin with Hapten-FITC. Total number of migratory (CD11c^+^MHC-II^hi^FITC^+^) DCs in the draining Lymph Nodes (LNs) of **(C)** Slamf8^-/-^ mice or **(D)** Slamf1^-/-^ mice, 24 h after FITC painting. Values represent mean. The data are representative of three or more independent experiments.

Innate immune cell trafficking is a critical process in triggering specific immune responses. For instance, inflammation of the skin induced by the hapten FITC leads to activation of skin dendritic cells (DCs) and their subsequent migration to the draining lymph nodes, which is primarily mediated by CC chemokine receptor 7 (CCR7) signaling [22]. A skin contact sensitization model was employed to assess whether migration of Slamf8^-/-^, Slamf1^-/-^ and wt DCs differed. To this end, the dorsal skin of mice was shaved and painted with 4 mg FITC overnight and cells from the draining lymph nodes were isolated. Migratory DCs were quantitated by flow cytometry by gating for CD11c^+^ MHC^hi^ FITC-positive cells (Fig. 1A) [23]. Compared to wt mice, the percentage and total number of FITC^+^ migratory DCs were significantly higher in Slamf8^-/-^ mice (Fig. 1B and 1C). As a difference in the number of dermal DCs may be responsible for the increased number of migrating DCs, the numbers of DCs in the skin of wt and Slamf8^-/-^ mice were compared. The number of CD11c^+^ cells in the skin of the ear were comparable in wt and Slamf8^-/-^ mice (S1A Fig.), showing that there is no numerical difference in the dermal DC population between wt and Slamf8^-/-^ mice [24]. By contrast, fewer FITC^+^-migratory DCs were detected in the draining lymph nodes of Slamf1^-/-^ mice (Fig. 1D), which was consistent with our previous finding that infiltration of Slamf1^-/-^ monocyte-derived mononuclear phagocytes into the inflamed lamina propria of colitic mice is impaired [17]. As there was no obvious difference in expression of CCR7 on the surface of skin DCs before or after FITC painting of wt and Slamf8^-/-^ mice (S1B and S1C Fig.), the altered DC migration might be due to a mechanism in addition to CCR7 signaling—the major contributor of DC migration to lymph nodes. Taken together, the outcomes of these studies indicate that the cell surface receptors Slamf1 and Slamf8 control *in vivo* migration of DCs in an opposite manner.

### Slamf8 negatively regulates *in vivo* migration of macrophages to the inflamed peritoneal cavity

**Fig 2:**
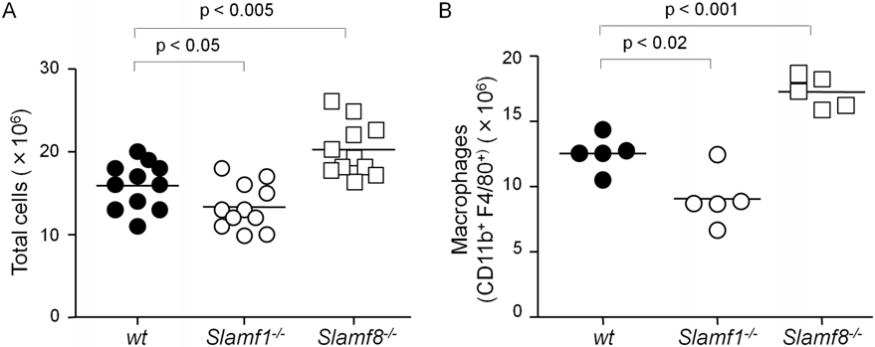
Slamf8 negatively and Slamf1 positively affects macrophage migration upon peritonitis induction. **(A)** Total number of peritoneal cells from wt, Slamf1^-/-^ and Slamf8^-/-^ *Balb/c* mice four days after *i*.*p*. injection of 4% thioglycollate. **(B)** Number of macrophages (CD11b^+^ F4/80^+^) among collected peritoneal cells. The data are representative of 3 independent experiments, each consisting of at least 3 mice per experimental condition.

Intraperitoneal injection [*i*.*p*.] of thioglycollate broth induces peritonitis in mice resulting in infiltration of different inflammatory cells in a time-dependent fashion [25]. Whereas neutrophils are the major infiltrating cells in the peritoneal cavity after 4 hours, macrophages begin to appear after 24 hours and become the major constituents after 48 hours. As shown in Fig. 2A, the total number of cells in the peritoneal cavity of Slamf1^-/-^ *Balb/c* mice was lower than the number of cells in wt *Balb/c* mice four days after induction of peritonitis. However, the number of cells in the peritoneal cavity of Slamf8^-/-^ *Balb/c* mice was increased as compared to wt mice (Fig. 2A). Consistent with the latter observation, four days after the injecting thioglycollate the number of CD11b^+^ F4/80^+^ macrophages in the peritoneum of Slamf8^-/-^ mice was higher than in the cavity of wt *Balb/c* mice (Fig. 2B). The number of macrophages in the peritoneal cavity of Slamf1^-/-^ *Balb/c* mice was lower than in wt *Balb/c* mice (Fig. 2B), confirming our previous observation assessing macrophages in Slamf1^-/-^ *C57BL/6J* mice [17]. We conclude that Slamf8^-/-^ macrophages display a stronger migration response to the induction of peritonitis. This supports the concept that Slamf8 plays an inhibitory role in migration of both macrophages and dendritic cells.

### Accelerated migration of macrophages and monocytes in the villi of the small intestine of Slamf8^-/-^ mice

**Fig 3:**
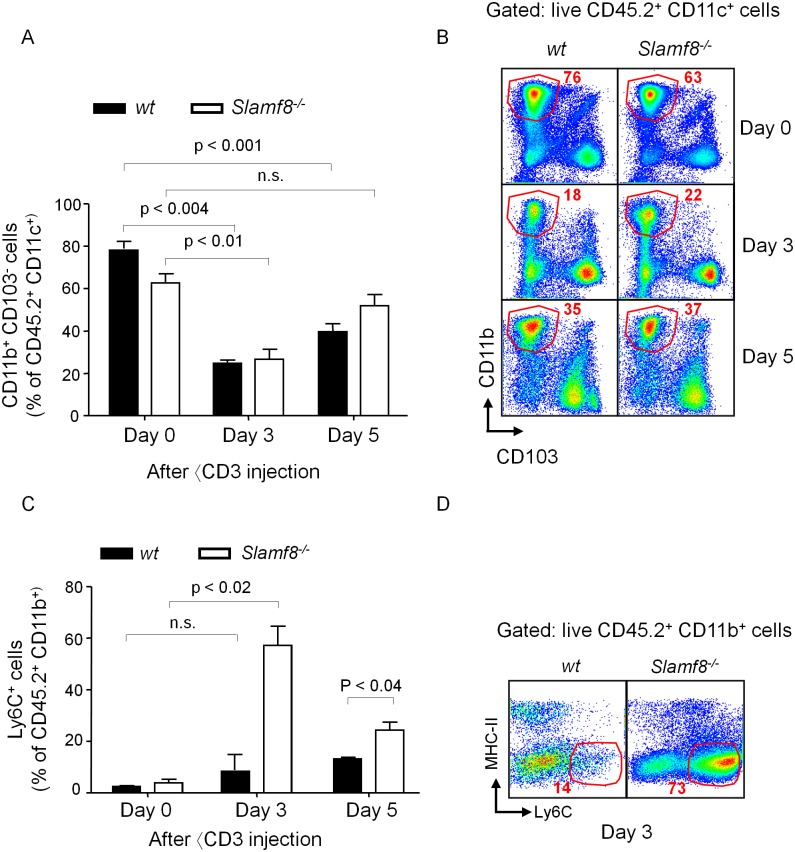
Slamf8 deficient mice show expedited macrophage repopulation of small intestine lamina propria after anti-CD3 mediated depletion. Depletion of lamina propria macrophages 3 and 5 days after administration of αCD3. **(A)** The percentage of CD11b^+^ CD103^-^ of total CD45.2^+^ CD11c^+^ Ly6G^-^ lamina propria cells. **(B)** Representative dot plots (gated on CD45.2^+^ CD11c^+^ Ly6G^-^) showing macrophages in lamina propria isolated cells from wt (left) and Slamf8^-/-^ (right) naïve mice (day 0) or injected with αCD3 3 days prior (middle), or 5 days prior (bottom). **(C)** Percentages of monocytes (MHC-II^low/-^ Ly6C^+^) of total CD11b^+^ CD45.2^+^ that were isolated from the lamina propria on day 0, 3, or 5 after αCD3 administration. **(D)** Representative dot plots showing monocytes in the lamina propria of the small intestine of wt and Slamf8^-/-^ mice on day 3. The data are representative of 2 independent experiments, each consisting of at least 3 mice per experimental condition.

To confirm the notion of an accelerated *in vivo* migration of Slamf8^-/-^ myeloid derived phagocytes we used a novel method by which repopulation of CX_3_CR1^+^ phagocytes in the villi of the small intestine is evaluated. Administration of αCD3 mAb to *C57BL/6J* mice activates cytotoxic CD8 T cells in the small intestine, which results in a transient depletion of CX_3_CR1^+^ phagocytes. Migratory monocytes then repopulate the villi of the small intestine [26]. As shown in Fig. 3A and 3B, three days after administering αCD3 to *Balb/c* mice a significant depletion of the number and percentage of CD11b^+^ CD11c^+^ CD45.2^+^ CD103^-^ mononuclear phagocytes in the small intestine was observed. The majority of these cells are CX_3_CR1^+^ macrophages of monocytic origin that reside in the small intestine [27, 28]. While Slamf8^-/-^ mice appear to have a slightly reduced percentage of intestinal macrophages in homeostatic conditions, their repopulation is significantly faster than that of wt mice. Three and five days post αCD3 injection, the CD11b^+^ CD11c^+^ CD45.2^+^ CD103^-^ cells begin to re-appear, more rapidly in Slamf8^-/-^ than in Slamf8^+/+^ mice. Slamf8^-/-^ mice show no statistical difference in the percentage between day 0 and day 5, but Slamf8^+/+^ mice have a lower percentage of these cells at day 5 (Fig. 3A). Surprisingly, a steep increase in CD11b^+^ Ly6C^+^ cells was observed in Slamf8^-/-^ mice three days after the last αCD3 injection compared to wt mice (Fig. 3C and 3D). This population, which was reduced in size at 5 days post injection, is known to represent newly arrived monocytes in the small intestine, which quickly transition to mature macrophages [29]. The results indicate that Slamf8^-/-^ mononuclear phagocyte precursors infiltrate the lamina propria at a faster rate than their wt counterparts supporting the notion that Slamf8 negatively regulates the migratory capacity of myeloid cells to immunologically active sites.

### Slamf8 negatively regulates Nox2 activity and *in vivo* migration of neutrophils

**Fig 4:**
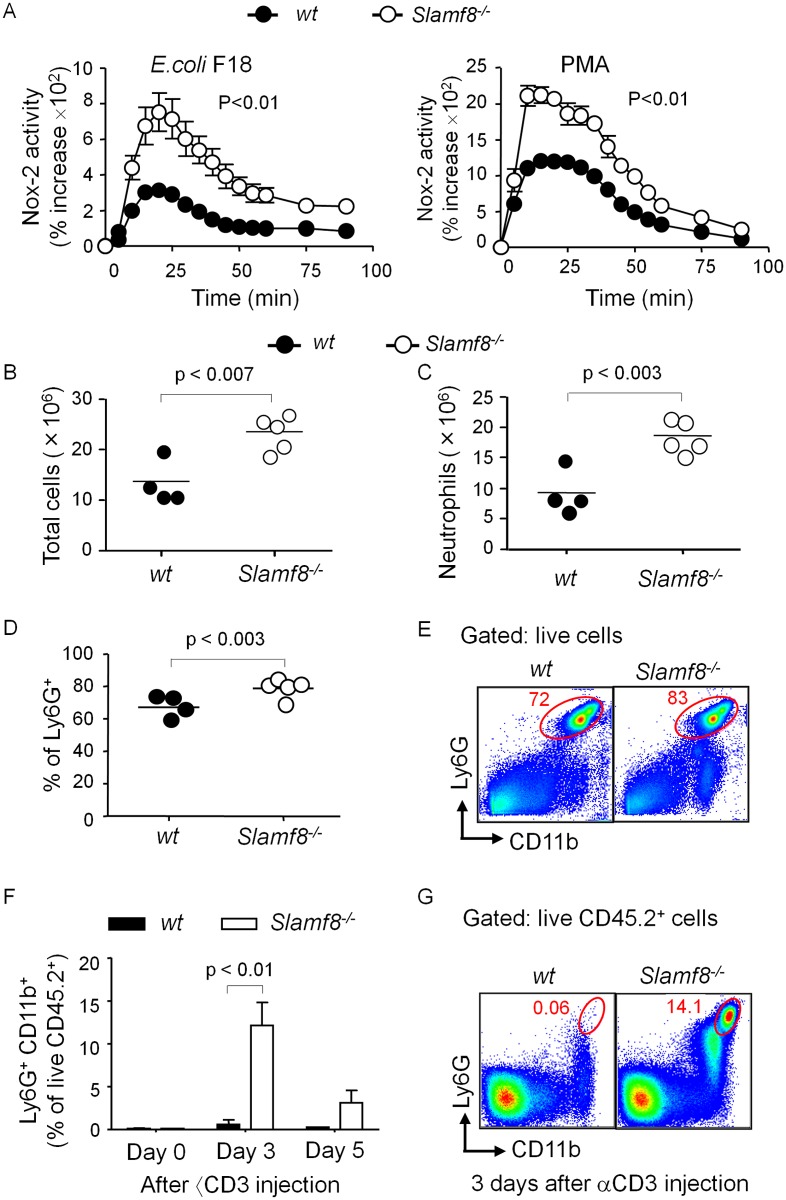
Slamf8^-/-^ neutrophil produce higher ROS, and Slamf8 negatively regulates neutrophil migration. **(A)** ROS production as measured by luminescence in wt and Slamf8^-/-^ mice thio-neutrophils in response to heat inactivated *E*.*coli* F18 and PMA. **(B)** Total number of peritoneal cells obtained from wt and Slamf8^-/-^ mice after 4 hours *i*.*p*. injection of 4% thioglycollate. **(C)** Number and (**D**) percentage of neutrophil (CD11b^+^ Ly6G^+^) among peritoneal cells in wt and Slamf8^-/-^ mice. **(E)** Representative dot plots (gated on DAPI^-^ cells) showing CD11b^+^ Ly6G^+^ neutrophil in the peritoneal cavity of wt and Slamf8^-/-^ mice. **(F**) Slamf8^-/-^ and wt mice were injected with αCD3. Percentage of neutrophils (CD11b^+^ Ly6G^high^) from the small intestine lamina propria 0, 3 and 5 days after αCD3 injection. **(G)** Representative dot plots (gated on CD45.2^+^) showing neutrophils. The data are representative of 2 independent experiments, each consisting of at least 3 mice per experimental condition.

Neutrophils, which migrate from the blood to a site of inflammation, represent the first line of defense against bacteria. Because we found a very high Nox2 activity in Slamf8^-/-^ macrophages and DCs (S2 Fig.) [10], we tested the ROS production by Slamf8^-/-^ neutrophils. As judged by a lucigenin-based assay, Slamf8^-/-^ neutrophils produce indeed significantly more ROS than wt *Balb/c* neutrophils in response in response to *E*. *coli* or to PMA (Fig. 4A).

We next evaluated the role of Slamf8 in neutrophil migration into the peritoneal cavity of wt and Slamf8^-/-^ mice 4 hours after *i*.*p*. injection of thioglycollate. Consistent with the macrophage data obtained 4 days after administering thioglycollate (Fig. 2), the total number of cells obtained from the peritoneal cavity of Slamf8^-/-^ mice was greater than the number of cells obtained from wt mice (Fig. 4B). Importantly, the number of CD11b^+^ Ly6G^+^ peritoneal neutrophils in Slamf8^-/-^ mice was 2-fold higher that of wt mice (Fig. 4C-4E).

We also evaluated migration of neutrophils in the villi of the small intestine on day 3 and 5 after administering αCD3 to Slamf8^-/-^ and wt mice (Fig. 4F-4G). Whilst the percentage of neutrophils in the small intestine comprised ~14% of the CD45.2^+^ cells in Slamf8^-/-^ mice on day 3 post injection, the percentage of neutrophils was ~5% on day 5. By contrast, on day 3 after administering αCD3 neutrophils comprised ~1% of the CD45.2^+^ cells in the lamina propria of wt mice, whereas no neutrophils were detected on day 5. In conclusion, Slamf8 negatively affects migration of neutrophils in two *in vivo* models consistent with the altered migration of DCs and monocyte-derived macrophages in Slamf8^-/-^ mice.

### Altered *in vitro* migration of Slamf8- and Slamf1-deficient DCs

**Fig 5:**
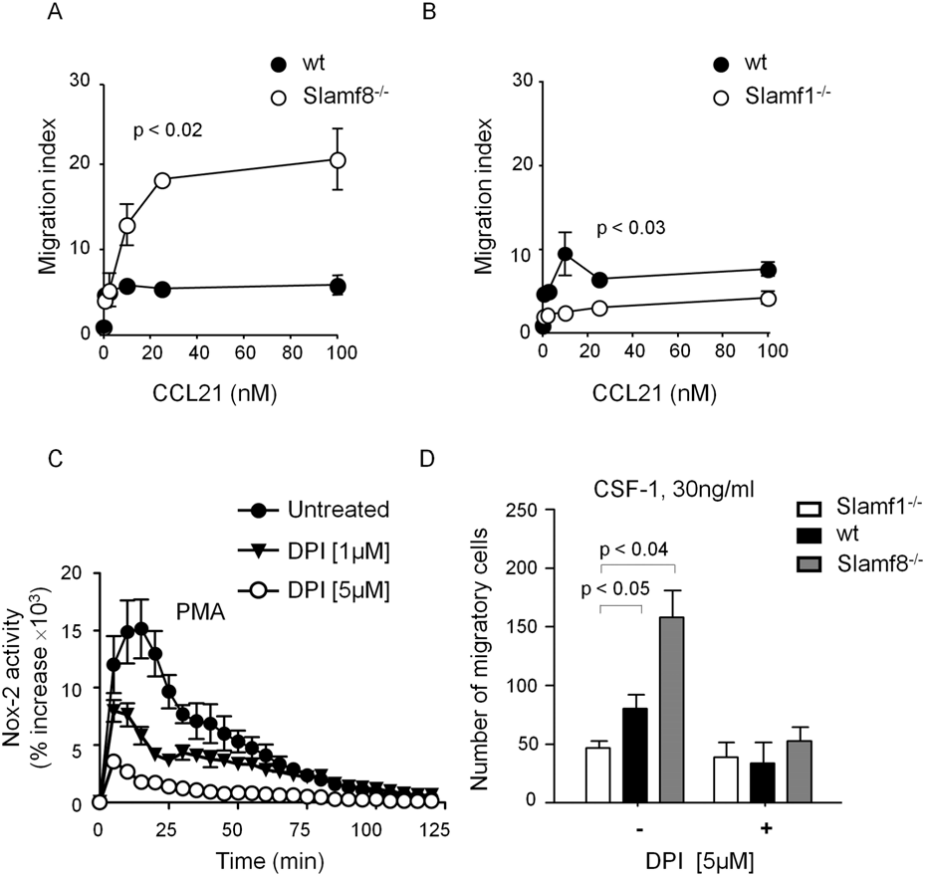
Slamf8 negatively regulates *in vitro* migration of DCs and of macrophages, while Slamf1 is a positive regulator of the same process. Wt, Slamf1^-/-^, and Slamf8^-/-^ skin DCs were isolated and column-purified before they were allowed to migrate toward a concentration range of CCL21 [0–400nM]. The migration index of **(A)** Slamf8^-/-^ vs. wt DCs and **(B)** wt vs. Slamf1^-/-^ DCs are plotted. **(C)** Macrophages from wt mice were incubated with different concentrations of DPI (1μM and 5μM) for 15 minutes, and the Nox2 activity in macrophages was quantified upon PMA (1mg/mL) stimulation. The data are representative of two independent experiments. **(D)** Wt, Slamf1^-/-^, and Slamf8^-/-^ thio-macrophages migration in the presence of CSF-1, with and without pre-incubation with DPI. All the data are representative of three independent experiments.

To test the hypothesis that cell intrinsic functions of Slamf1 and Slamf8 give rise to an altered migratory capacity, chemotaxis was examined in a transwell migration assay were. We focused on CCR7 and its two major ligands, CCL21 and CCL19, which play an important role in facilitating migration of DCs in the lymphatic system and within lymph nodes [30]. DCs that were obtained by CD11c positive selection from the skin of the ear were allowed to migrate through 5μm pores in a transwell system toward CCL21 [0–400nM]. Slamf8^-/-^ migrated much more efficiently toward CCL21 than wt *Balb/c* DCs (Fig. 5A). *In vitro* trafficking of skin-derived Slamf8^-/-^ DCs in response to CCL19 was similarly increased (S3 Fig.). In contrast to Slamf8^-/-^ cells, migration of Slamf1^-/-^ skin-derived DCs toward CCL21 was markedly reduced (Fig. 5B). These differences in *in vitro* migration indicate that this process is DC-intrinsic. As the surface expression of CCR7 is similar between Slamf8^-/-^ and wt DCs the differences between wt and deficient DCs could not be due to altered expression of CCR7 (S1B and S1C Fig.).

A role for Nox2-dependent production of ROS in migration of bone marrow derived macrophages in response to the chemo-attractant CSF-1 was suggested by experiments in gp91^-/-^ mice, which lack an active Nox2 complex [15, 31]. To test the hypothesis that cell intrinsic functions of Slamf1 and Slamf8 affect cell migration in response to CSF-1 in a ROS-mediated fashion an *in vitro* transwell system was used in the absence and presence of the Nox2 inhibitor diphenyleneiodonium chloride (DPI), which effectively inhibits human neutrophil migration [14]. First, we determined that 5μM of DPI inhibited PMA induced Nox2 activity of peritoneal macrophages without affecting cell viability (Fig. 5C and S4 Fig.). In response to CSF-1, *in vitro* migration of Slamf1^-/-^ peritoneal macrophages was impaired, while an enhanced migration of Slamf8^-/-^ macrophages was observed (Fig. 5D). This observation is dependent on ROS as incubation with DPI 15 minutes before the cells were allowed to migrate toward CSF-1 dramatically reduced the number of migrating cells. Thus, cell intrinsic migration towards CSF-1 is altered in Slamf1^-/-^ and Slamf8^-/-^ macrophages. Additionally, this cannot be due to altered expression of the CSF-1 receptor CSF-1R (CD115), as shown in S5 Fig. Taken together, these data suggest the signaling of two key cell surface receptors CCR7 and CSF-1R is altered in the Slamf1- and Slamf8-deficient cells in a ROS-dependent fashion.

### The self-ligand Slamf8 affects the *in vivo* migration of DCs

**Fig 6:**
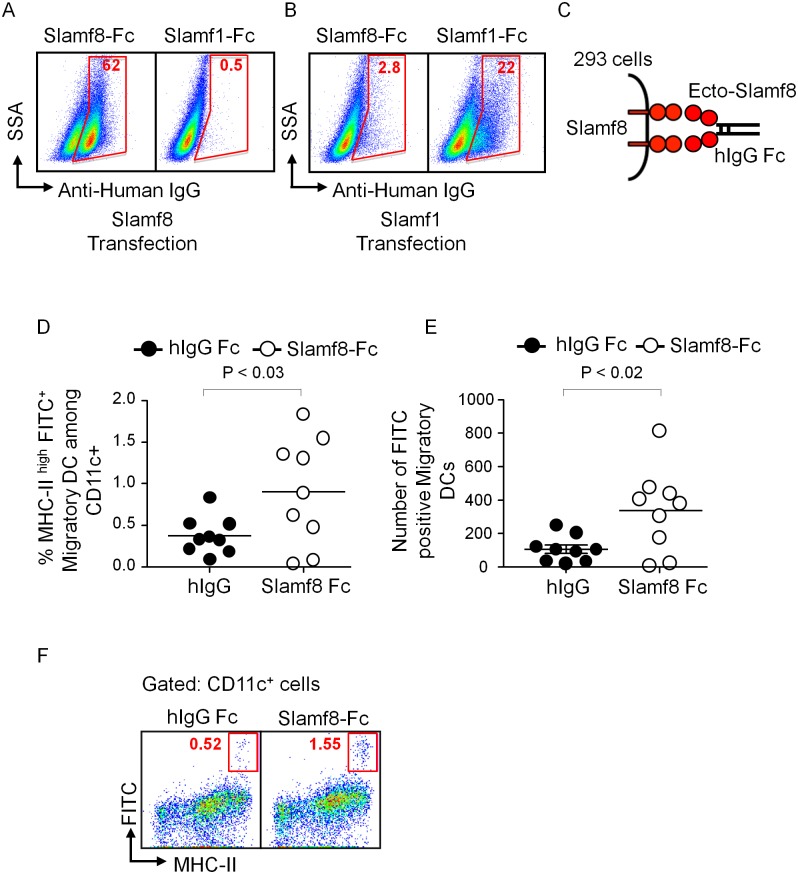
Slamf8 is a homophilic receptor, and Slamf8-Fc enhances DCs migration *in vivo*. Slamf8 receptor interacts in a homophilic manner. HEK 293 cells were transfected with Slamf8 **(A)** or Slamf1 **(B)**, and the binding of Slamf1-Fc and Slamf8-Fc to these transfected cells was determined by flow cytometry. **(C)** Scheme for the Slamf8-Fc binding assay. The data are representative of three independent experiments. Slamf8-Fc enhances DCs migration *in vivo*. Wt *Balb/c* mice were *i*.*p*. injected with Slamf8-Fc or human IgG1. Three hours later, FITC painting assay was performed. **(D)** Percentages among CD11c^+^ cells **(E)** total number of migratory (MHC-II^hi^ FITC^+^) DCs in the draining LNs. **(F)** Representative dot plots (gated on CD11c^+^ cells) showing migratory MHC-II^hi^ FITC^+^ DCs in the draining lymph nodes. The data are representative of two independent experiments.

The notion that majority of human and mouse Slamf adhesion molecules are homophilic prompted us to evaluate whether Slamf8 would be a self-ligand receptor. To this end, a soluble fusion protein comprised of the mouse Slamf8 ectodomain and the human IgG1 Fc domain (Slamf8-Fc) was generated. Next, 293 cells transiently transfected with Slamf8 or Slamf1 were incubated with the Slamf8-Fc protein and stained with a fluorescent antibody directed against the Fc portion of human IgG [32]. As predicted, Slamf8-Fc associated with Slamf8 transfected 293 cells, but not with Slamf1 transfectants. In the reciprocal experiment Slamf1-Fc associated with Slamf1 transfected cells but did not bind to Slamf8 transfected 293 cells (Fig. 6A-6C). Thus, Slamf8 is a homophilic receptor, which does not ligate *in trans* with Slamf1.

Next, the effect of Slamf8-Fc in the skin contact sensitization model was evaluated. To this end wt mice were injected with the Slamf8-Fc fusion protein 3 hours prior to painting 2 mg FITC onto the shaved skin. Mice that had received the Slamf8-Fc fusion protein both the percentage and the number of migratory DCs were significantly elevated compared to the mice injected with human IgG1 (Fig. 6D-6F). This suggests that Slamf8-Fc interrupted the homophilic Slamf8 interactions that suppress DC migration (Fig. 1).

## Discussion

Three distinct *in vivo* models, *i*.*e*. skin sensitization by painting with the hapten FITC, thioglycollate induced peritonitis and migration in the lamina propria of the small intestine, were employed to demonstrate that migration of Slamf8-deficient DCs, macrophages and neutrophils is increased. This indicates that Slamf8 negatively regulates this process in wt mice. *In vitro* transwell experiments showed an enhanced migration of Slamf8^-/-^ macrophages in response to CSF-1 and dendritic cells in response to the chemokines CCL19 and CCL21, which bind to the CCR7 receptor [30]. The increased migration of Slamf8^-/-^ macrophages and DCs is cell intrinsic and macrophage migration is inhibited by the Nox2 inhibitor DPI. We conclude that Nox2-dependent ROS production, which raises intracellular and extracellular levels of H_2_O_2_ causes accelerated migration of Slamf8^-/-^ phagocytes. To our knowledge, Slamf8-deficient mice are one of two mutant mice with an inducible increased ROS production, the other being the recently discovered Negative Regulator of ROS (NRROS) [33], which was studied in the context of autoimmune disease. In wt cells Slamf8 is therefore a negative regulator of signal transduction events in myeloid cells, which affect their migration by interacting with its self-ligand on endothelial cells or fibroblastic reticular cells. By contrast, Slamf1 appears to enhance migration of phagocytes by stimulating Nox2-dependent events. Hence, myeloid cell migration is consistently impaired in Slamf1^-/-^ mice, which generate low levels of ROS in response to several bacteria, LPS and other inflammation related biologicals [6, 17].

The classical phagocytic NADPH oxidase (Nox2) produces reactive oxygen upon assembly of the membrane bound catalytic gp91^phox^ and p22^phox^ with at least four cytosolic subunits p40^phox^, p47^phox^, p67^phox^, Rac1/2 [11]. Nox2 complex assembly is a tightly regulated process that involves phosphorylation of its cytosolic subunits or binding of p40^phox^ to the lipid phosphatidyl-3'-phosphate (PI_3_P), which is phosphorylated by the Class III phosphatidyl inositol kinase Vps34 [34]. The latter enzyme can be recruited by the intracellular tail of Slamf1 and in the absence of Slamf1 macrophages, dendritic cells, and neutrophils produce reduced amounts of ROS and consequently H_2_O_2_ [6, 7]. ROS in phagocytes, which plays a role in killing of some bacteria, is also involved in many aspects of innate immune responses, *e*.*g*. NF-κB signaling through TRAF6 recruitment [35]. Perhaps the best-known signaling effects of ROS are in oxidation of specific Cys residues of the protein tyrosine phosphatases SHP-1 and SHP-2, which impair their enzymatic function thus allowing for enhanced phosphorylation by tyrosine kinases [36, 37]. Another example of these phosphatases is the lipid phosphatase PTEN, which impact directional mobility [38]. Effects on cytoskeletal functions is exemplified by β-actin Cys374 that can be S-glutathionylated by ROS thereby promoting cell spreading, which is a necessary step in adequate migration [39]. DC migration was shown to be affected through the function of oxidative stress response kinase 1 (OSR-1) [40]. Direct evidence of the involvement of Nox2 in cell migration and chemotaxis comes from studies that used mice with genetic ablations of Nox2 components. For instance, Nox2 knock out (gp91^phox-/-^) bone marrow macrophages completely lost their chemotactic response toward a CSF-1 gradient, which is likely due to a reduction of ERK1/2 phosphorylation leading to defected cell migration [15]. In the murine macrophage cell line RAW264.7 Nox2 is required for LPS induced matrix metalloproteinase MMP-9 expression, which eventually influences cell migration [41]. In sum, ROS signaling has implications on a variety processes including migration, adhesion and chemotaxis.

Using a small-molecule screen for drugs capable of inhibiting neutrophil chemotaxis, Hattori and colleagues found that the Nox2 inhibitor diphenyleneiodonium chloride (DPI), can effectively inhibit human neutrophil directionality during migration. Furthermore, they found that both gp91^phox-/-^ murine neutrophil and neutrophil from Chronic granulomatous disease (CGD) patients lost their directional migration, establishing the notion that Nox2 activity influences cell migration [14].

Our previous study shows that expression of murine Slamf8 is not only significantly induced in macrophages by bacteria and bacterial components, *e*.*g*. *E*. *coli* and LPS, but more dramatically by IFN-γ [10]. As shown in S6A–D Fig., IFN-γ also induces Slamf8 transcripts in bone marrow derived DCs, peritoneal neutrophils, human monocytes and macrophages and protein expression in peritoneal macrophages. Perhaps more importantly, Slamf8 expression can be induced by TNF-α treatment of mouse heart endothelial cells (S6E Fig.), consistent with the notion that the homophilic interaction might play a role in cell migration. That concept is also supported by the observation that Slamf8 is expressed in skin and mesenteric lymph nodes by fibroblastic reticular cells (FRC) [18, 42], which function as a scaffold for the support and migration of hematopoietic cells [43].

**Fig 7:**
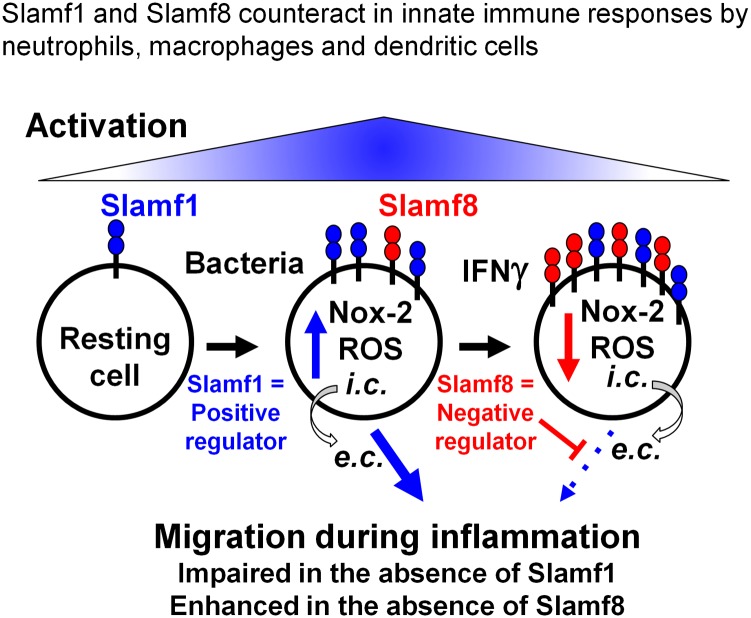
Proposed model showing Slamf1 and Slamf8 are counterparts for the regulation of Nox2 generated ROS and cell migration. In migratory phagocytes, the early actor Slamf1 enhances ROS-mediated migration signals; subsequently Slamf8 reduces ROS-mediated migration signals. Inflammatory signals enhance the activity of Slamf1, which induces Nox-2 mediated ROS production. Inflammatory mediators, such as IFN-γ subsequently increase Slamf8 expression and function. This leads to the suppression of ROS production resulting in the reduction in infiltration of phagocytes. Thus, Slamf1 and Slamf8 together, balance the extent of infiltration of inflammatory cells.

Based upon this study and published work employing Slamf1 as well as Slamf8 deficient mice and mutant macrophage cell lines [5–7, 10, 17], we propose a model in which Slamf1 and Slamf8 help shape innate immune responses (Fig. 7). This includes differential expression and induction of Nox2 activity in response to innate stimuli [6, 10]. Furthermore, the model graphically represents how migration of various myeloid cells is dependent upon the two receptors. Initial inflammatory queues may trigger Slamf1-meditated Nox2 activation resulting in a spike in ROS, which is rapidly converted into hydrogen peroxide that contributes to the migratory activity of Slamf1-expressing cells. Subsequent inflammatory mediators (IFN-γ) induce Slamf8 expression, which suppresses the production of ROS and hence reduces additional migratory infiltration.

Taken together, the outcomes of our studies provide the first evidence that Slamf8 is a novel homophilic receptor, and that Slamf1 and Slamf8 oppositely regulate macrophage migration in a Nox2 dependent manner. The similarity of the *in vivo* migration and *in vitro* ROS production phenotypes suggest that Nox2 has a similar involvement in the migration of DCs and neutrophils as macrophages. These findings point out that Slamf1 and Slamf8 are potential therapeutic targets to modulate DC, macrophage and neutrophils migration and function, thus Slamf1 and Slamf8 based therapeutic strategies can be pursued to regulate cellular immune response. As Slamf8^-/-^ mice show an enhanced migration of antigen presenting cells and Slamf8-Fc approximates the Slamf8^-/-^ phenotype, Slamf8 may be of interest for potential therapeutic applications in vaccine development.

## Supporting Information

S1 Fig Comparable skin DC numbers and CCR7 expression in wt and Slamf8^-/-^ DCs. **(A)** Both Ears were collected from wt and Slamf8^-/-^ mice, the ear skin was digested with DNase and Liberase, the single cell suspensions were obtained. The CD11c^+^ cells were further isolated using CD11c positive selection column MicroBeads (Miltenyi Biotec) and the individual number of CD11c+ cells from each mouse was quantified. The data are representative of 5 independent experiments, each consisting of at least 5 mice per experimental condition. **(B)** Flow cytometric representation of wt and Slamf8^-/-^ CCR7 expression in CD11c^+^ cells isolated from naïve mouse ear skin. **(C)** 24 hours after administration of FITC on the mouse dorsal skin, wt and Slamf8^-/-^ CCR7 expression in the migratory DCs (CD11c^+^ MHC-II^high^ FITC^+^) in skin draining lymph nodes.
(TIF)

S2 Fig Slamf8^-/-^ macrophages produce more intracellular ROS than wt macrophages. Wt and Slamf8^-/-^ mice thio-macrophages were incubated with CM-H_2_DCFDA for 1 hour, and then stimulated with heat inactivated *E*.*coli* F18 for 2 hours. The intracellular ROS generation was quantified by flow cytometry. Representative histogram shows an enhanced intracellular ROS production in Slamf8^-/-^ macrophages at the 30-minute time point after stimulation.
(TIF)

S3 Fig Slamf8^-/-^ DCs migrate more efficiently toward CCL19. Wt and Slamf8^-/-^ skin DCs were isolated and column-purified before they were allowed to migrate toward a concentration range of CCL19 [0–400nM]. The relative migration of wt and Slamf8^-/-^ DCs is plotted as the migration index.
(TIF)

S4 Fig DPI (5μM) incubation does not affect cell viability. Thio-macrophages were incubated without or with DPI [1 and 5μM] for 3 hours in complete RPMI. The cell viability was determined by staining with DAPI. The percentage of DAPI+ (dead) cells was quantified by flow cytometry.
(TIF)

S5 Fig Comparable CD115 expression in wt, Slamf1^-/-^, and Slamf8^-/-^ macrophages. CSF-1R (CD115) expression in wt, Slamf1^-/-^, and Slamf8^-/-^ thio-macrophages (CD11b^+^ F4/80^+^) assessed by flow cytometry.
(TIF)

S6 Fig Slamf8 expression can be induced by inflammatory stimulation. Slamf8 mRNA expression in **(A)** thio-neutrophils and **(B)** bone marrow DCs upon overnight IFN-γ (10ng/mL) activation was quantified by Taqman. **(C)** Expression of human SLAMF8 by purified PBMC monocytes, before and after differentiation into macrophages (M-CSF, GM-CSF) and after stimulation with various inflammatory mediators (IFN-γ, LPS). **(D)** Thio-macrophages from wt and Slamf8^-/-^ mice were stimulated with IFN-γ (10ng/ml) plus LPS (100ng/ml) overnight. Slamf8 protein expression was detected with anti-Slamf8 polyclonal antibody (R&D System) by Western Blot. **(E)** Mouse vascular endothelial cells were isolated from new born mice heart and treated with TNF-α (100ng/mL) for 24 hours. Slamf8 transcripts were quantified by Taqman. The results were normalized to the expression of the house keeping gene 18SrRNA and presented relative to untreated cells. The data are representative of 2 independent experiments.
(TIF)
