# Supplementary figures and images for: Migration of Myeloid Cells during Inflammation Is Differentially Regulated by the Cell Surface Receptors Slamf1 and Slamf8
Source: PLoS One. 2015 Mar 23;10(3):e0121968. doi: 10.1371/journal.pone.0121968 (PMC4370648; doi:10.1371/journal.pone.0121968)

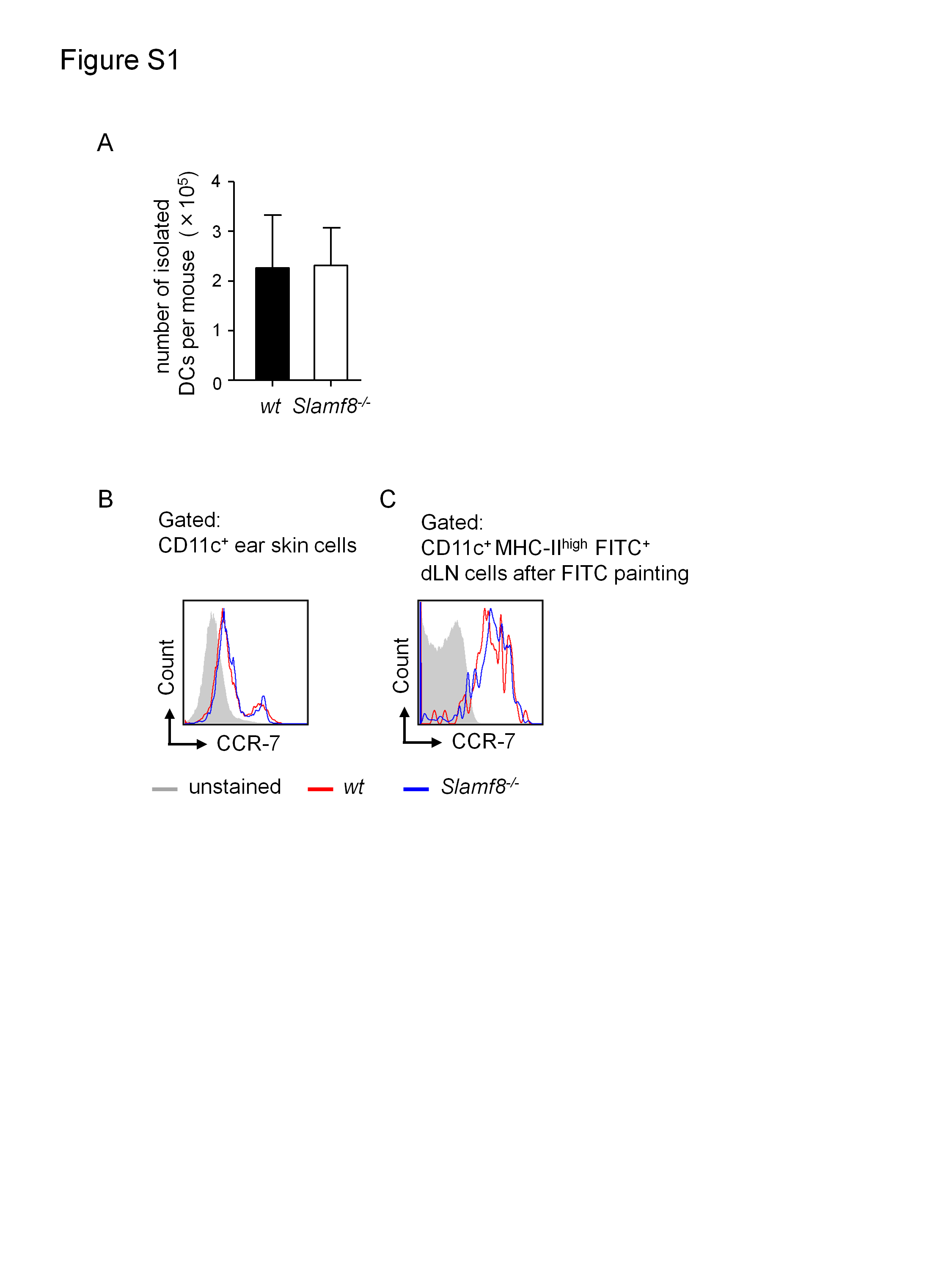

Supplement: S1 Fig — (A) Both Ears were collected from wt and Slamf8-/- mice, the ear skin was digested with DNase and Liberase, the single cell suspensions were obtained. The CD11c+ cells were further isolated using CD11c positive selection column MicroBeads (Miltenyi Biotec) and the individual number of CD11c+ cells from each mouse was quantified. The data are representative of 5 independent experiments, each consisting of at least 5 mice per experimental condition. (B) Flow cytometric representation of wt and Slamf8-/- CCR7 expression in CD11c+ cells isolated from naïve mouse ear skin. (C) 24 hours after administration of FITC on the mouse dorsal skin, wt and Slamf8-/- CCR7 expression in the migratory DCs (CD11c+ MHC-IIhigh FITC+) in skin draining lymph nodes. (TIF) [file pone.0121968.s001.TIF]

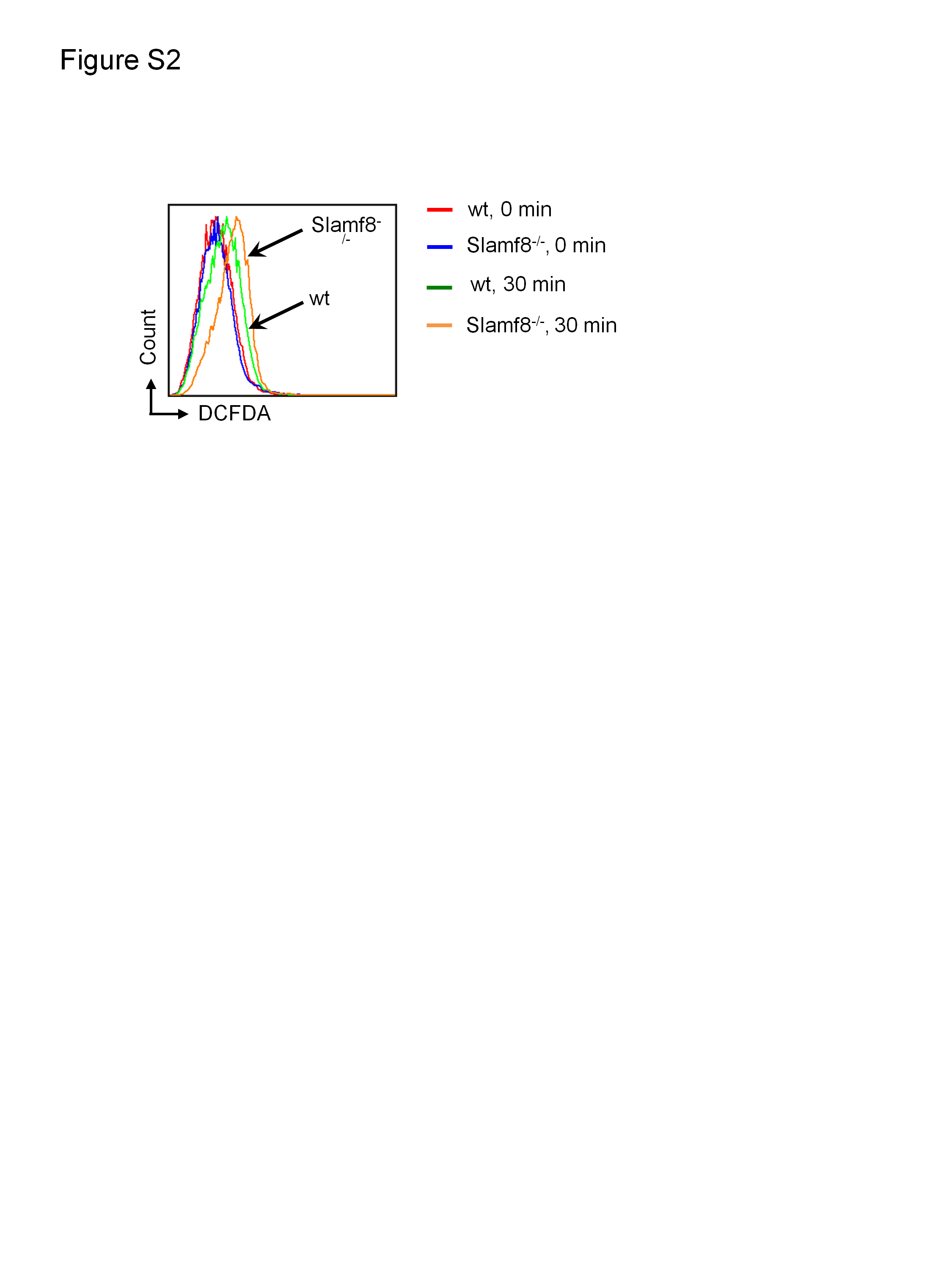

Supplement: S2 Fig — Wt and Slamf8-/- mice thio-macrophages were incubated with CM-H2DCFDA for 1 hour, and then stimulated with heat inactivated E.coli F18 for 2 hours. The intracellular ROS generation was quantified by flow cytometry. Representative histogram shows an enhanced intracellular ROS production in Slamf8-/- macrophages at the 30-minute time point after stimulation. (TIF) [file pone.0121968.s002.TIF]

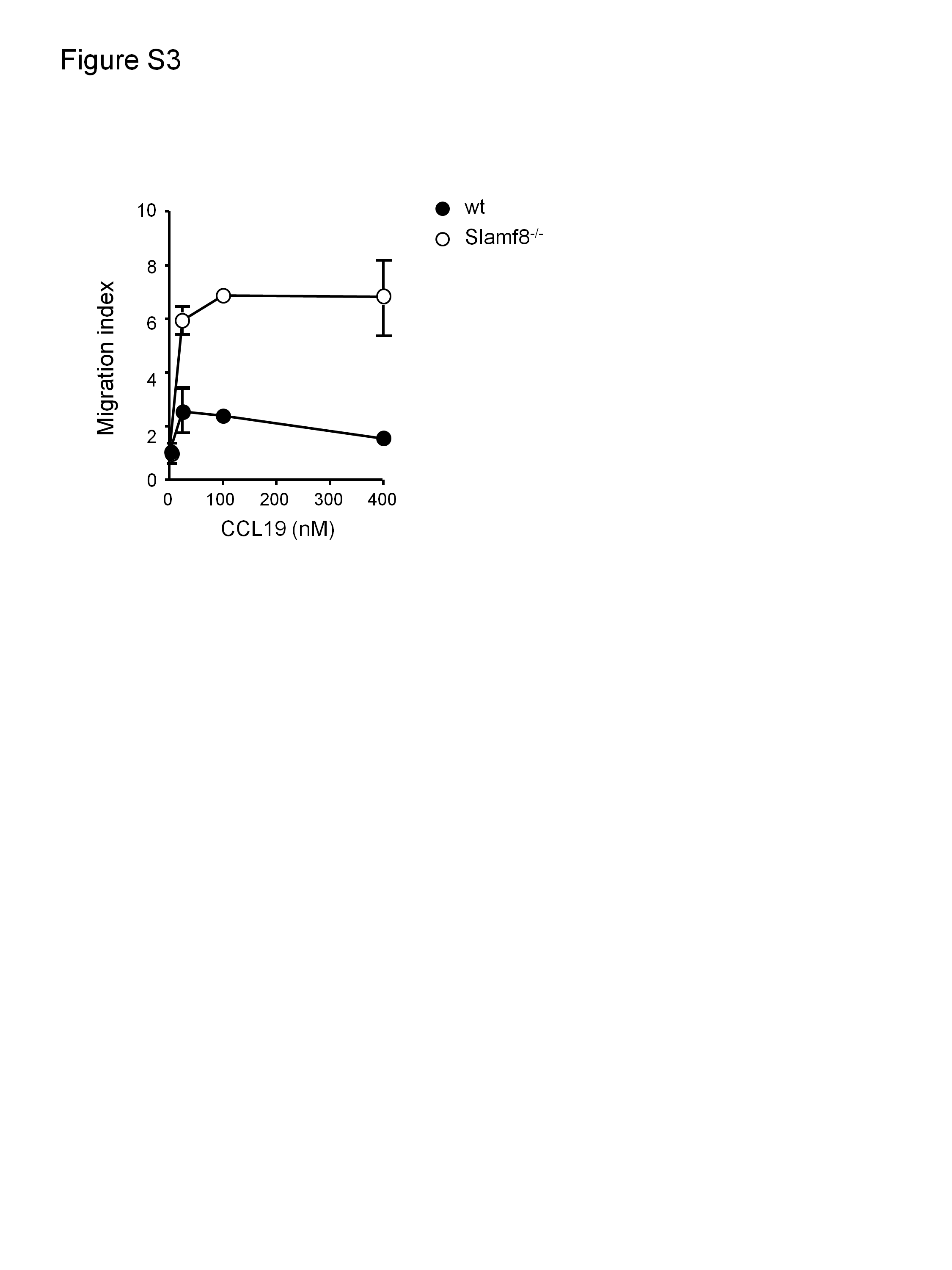

Supplement: S3 Fig — Wt and Slamf8-/- skin DCs were isolated and column-purified before they were allowed to migrate toward a concentration range of CCL19 [0–400nM]. The relative migration of wt and Slamf8-/- DCs is plotted as the migration index. (TIF) [file pone.0121968.s003.TIF]

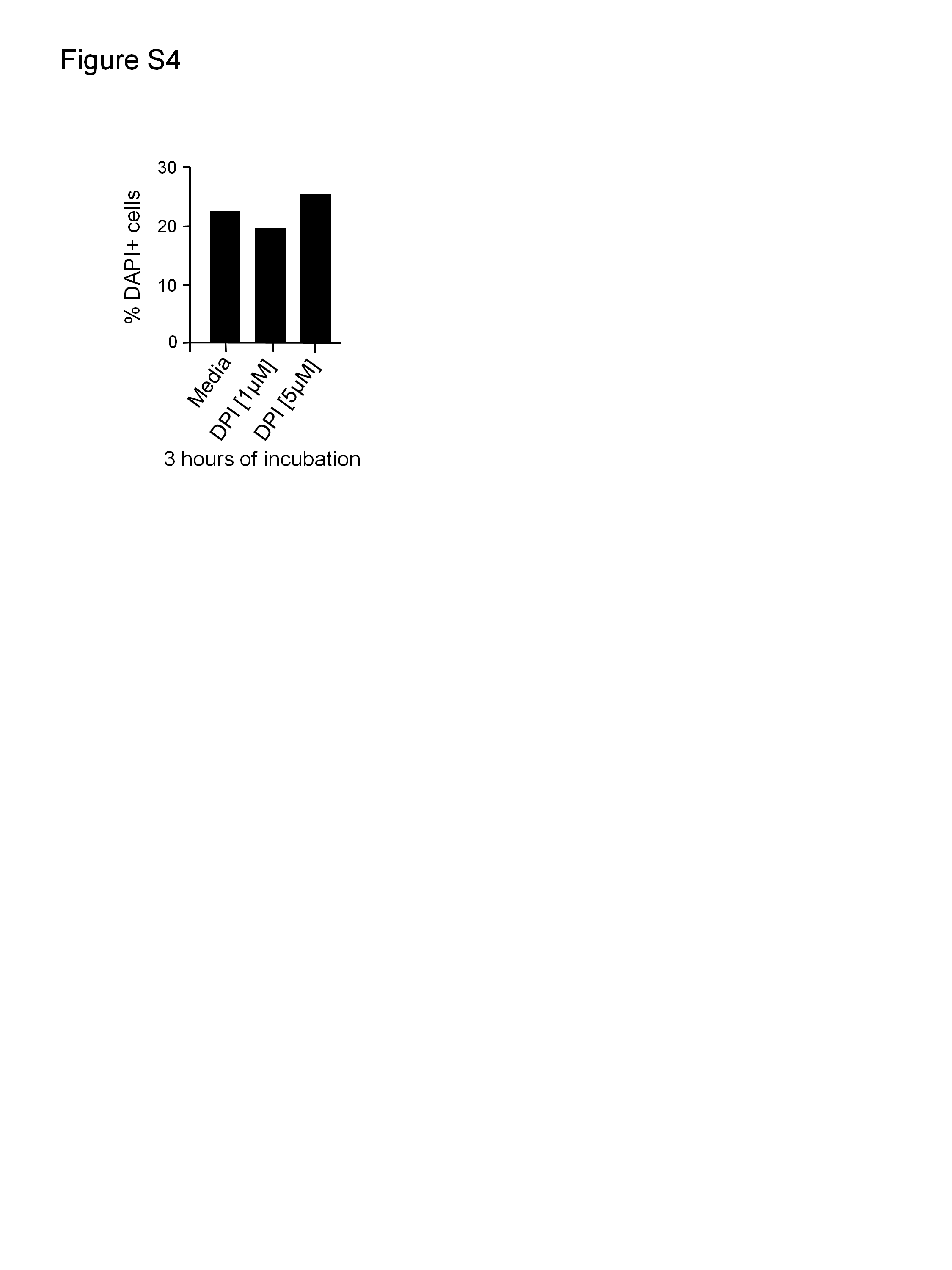

Supplement: S4 Fig — Thio-macrophages were incubated without or with DPI [1 and 5μM] for 3 hours in complete RPMI. The cell viability was determined by staining with DAPI. The percentage of DAPI+ (dead) cells was quantified by flow cytometry. (TIF) [file pone.0121968.s004.TIF]

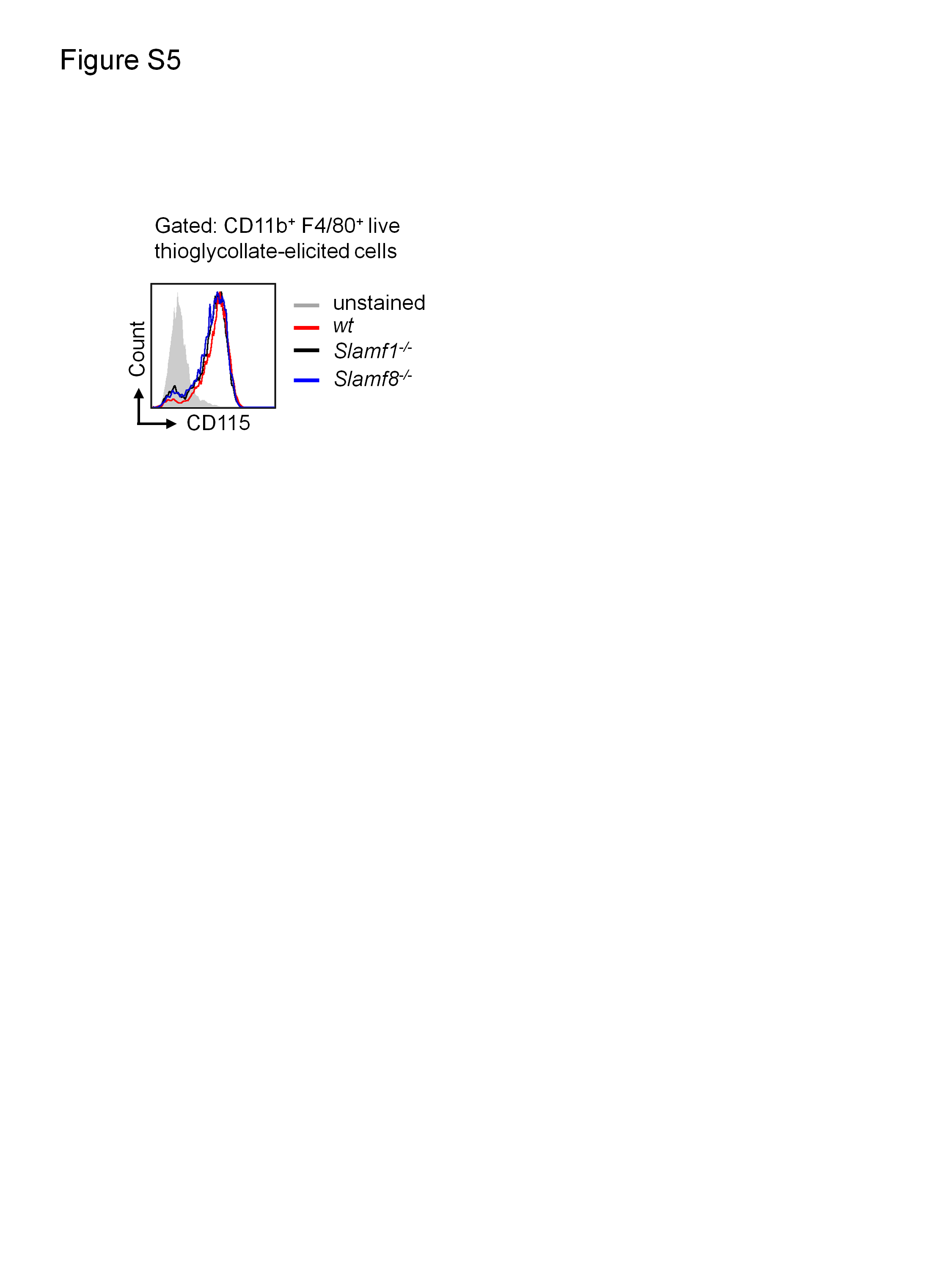

Supplement: S5 Fig — CSF-1R (CD115) expression in wt, Slamf1-/-, and Slamf8-/- thio-macrophages (CD11b+ F4/80+) assessed by flow cytometry. (TIF) [file pone.0121968.s005.TIF]

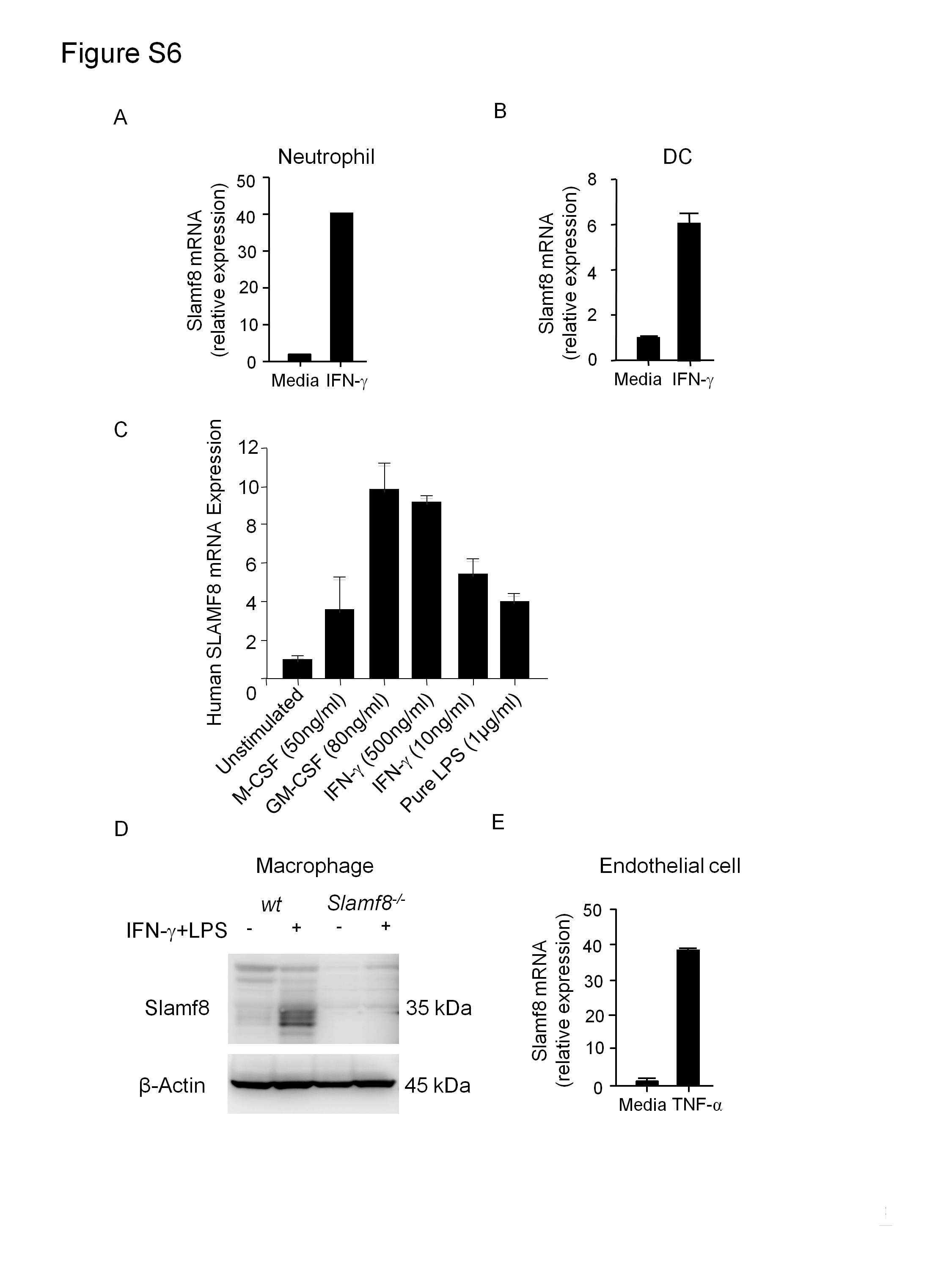

Supplement: S6 Fig — Slamf8 mRNA expression in (A) thio-neutrophils and (B) bone marrow DCs upon overnight IFN-γ (10ng/mL) activation was quantified by Taqman. (C) Expression of human SLAMF8 by purified PBMC monocytes, before and after differentiation into macrophages (M-CSF, GM-CSF) and after stimulation with various inflammatory mediators (IFN-γ, LPS). (D) Thio-macrophages from wt and Slamf8-/- mice were stimulated with IFN-γ (10ng/ml) plus LPS (100ng/ml) overnight. Slamf8 protein expression was detected with anti-Slamf8 polyclonal antibody (R&D System) by Western Blot. (E) Mouse vascular endothelial cells were isolated from new born mice heart and treated with TNF-α (100ng/mL) for 24 hours. Slamf8 transcripts were quantified by Taqman. The results were normalized to the expression of the house keeping gene 18SrRNA and presented relative to untreated cells. The data are representative of 2 independent experiments. (TIF) [file pone.0121968.s006.tif]
